# Associations between parental rules, style of communication and children’s screen time

**DOI:** 10.1186/s12889-015-2337-6

**Published:** 2015-10-01

**Authors:** Mona Bjelland, Bart Soenens, Elling Bere, Éva Kovács, Nanna Lien, Lea Maes, Yannis Manios, George Moschonis, Saskia J te Velde

**Affiliations:** Department of Nutrition, Faculty of Medicine, University of Oslo, P.O. Box 1046 Blindern, NO-0316 Oslo, Norway; Department of Developmental, Personality and Social Psychology, Faculty of Psychology and Educational Sciences, Ghent University, Ghent, Belgium; Department of Public Health, Sport and Nutrition; Faculty of Health and Sport Sciences, University of Agder, Kristiansand, Norway; Department of Paediatrics, Faculty of Medicine, University of Pécs, Pécs, Hungary; Institute for Medical Information Processing, Biometrics and Epidemiology and German Center for Vertigo and Balance Disorders, Ludwig Maximilian University, Munich, Germany; Department of Public Health, Ghent University, Ghent, Belgium; Department of Nutrition and Dietetics, Harokopio University, Athens, Greece; Department of Epidemiology & Biostatistics and the EMGO Institute for Health & Care Research, VU University Medical Center, Amsterdam, The Netherlands

**Keywords:** Children, Parents, Sedentary, Television, Computer, Social control

## Abstract

**Background:**

Research suggests an inverse association between parental rules and screen time in pre-adolescents, and that parents’ style of communication with their children is related to the children’s time spent watching TV. The aims of this study were to examine associations of parental rules and parental style of communication with children’s screen time and perceived excessive screen time in five European countries.

**Methods:**

UP4FUN was a multi-centre, cluster randomised controlled trial with pre- and post-test measurements in each of five countries; Belgium, Germany, Greece, Hungary and Norway. Questionnaires were completed by the children at school and the parent questionnaire was brought home. Three structural equation models were tested based on measures of screen time and parental style of communication from the pre-test questionnaires.

**Discussion:**

Of the 152 schools invited, 62 (41 %) schools agreed to participate. In total 3325 children (average age 11.2 years and 51 % girls) and 3038 parents (81 % mothers) completed the pre-test questionnaire. The average TV/DVD times across the countries were between 1.5 and 1.8 h/day, while less time was used for computer/games console (0.9–1.4 h/day). The children’s perceived parental style of communication was quite consistent for TV/DVD and computer/games console. The presence of rules was significantly associated with less time watching TV/DVD and use of computer/games console time. Moreover, the use of an autonomy-supportive style was negatively related to both time watching TV/DVD and use of computer/games console time. The use of a controlling style was related positively to perceived excessive time used on TV/DVD and excessive time used on computer/games console. With a few exceptions, results were similar across the five countries.

**Conclusions:**

This study suggests that an autonomy-supportive style of communicating rules for TV/DVD or computer/ games console use is negatively related to children’s time watching TV/DVD and use of computer/games console time. In contrast, a controlling style is associated with more screen time and with more perceived excessive screen time in particular. Longitudinal research is needed to further examine effects of parental style of communication on children’s screen time as well as possible reciprocal effects.

**Trial registration:**

International Standard Randomized Controlled Trial Number Register, registration number: ISRCTN34562078. Date applied29/07/2011, Date assigned11/10/2011.

## Background

Sedentary behaviour is defined primarily as sitting behaviours such as TV viewing and computer use, representing a slight increase in expenditure above resting metabolic rate, but below the expenditure seen in light-intensity physical activity [1, 2]. Time spent sedentary, overall and in front of screens, is negatively associated with physical health [1]. Despite the unfavourable health outcomes derived from increased sedentary activity levels (5–10 h/day), many children engage in more than 2 h of screen-based behaviours per day [1–4]. In order to discourage time spent sedentary, more knowledge is needed about factors which determine or are related to these sedentary behaviours and overall sedentary time. Previous research has shown that individual factors (gender and age), demographic factors (socio-economic status) and family environmental factors such as availability of TVs, parental modelling and parental rules, are related to overall sedentary time and/or screen time [1, 2, 5, 6]. Age is also an important factor in a prevention perspective. The age of 10–11 years is called a “key transition age” [7] because adolescents are establishing behavioural patterns that may continue into adulthood and have implications for long term health. Yet behaviours are more easily changed or prevented when they are still being developed or recently established than when part of a lifestyle [8].

In particular, presence of parental rules and style of communication are two important family environmental factors. Several studies have found an inverse association between parental rules and screen time in pre-adolescents, reported either by children or their parents [9–14]. Further research exploring rules related to regulation of TV/DVD and computer/game console activities is warranted, as well as studies investigating barriers and facilitators to reduce screen time within the family home environment – from both the child and parent perspective [15]. Furthermore, research suggests that parents’ style of communication with their children is related to the children’s time spent watching TV [16, 17]. Jago et al. [16] explored to what extent parenting styles and practices are associated with children’s TV viewing, but emphasize that they did not examine the relative efficacy of different approaches of communicating restriction messages. The authors conclude “Therefore, future work needs to focus on how best to deliver restriction messages….” (p. 576).

In this study, parents’ style of communicating rules was conceptualized on the basis of several theories on parental socialization, in particular Hoffman’s theory of moral development [18] and Self-Determination Theory (SDT) [19]. Hoffman [18] distinguished between several styles of introducing parental rules, the most important ones of which are inductive discipline (i.e., pointing out the consequences of the child’s behaviour) and power assertion (i.e., forceful control of the child’s behaviour through harsh behaviours such as physical punishment or the threat of punishment). Research has shown that inductive discipline is more effective in fostering children’s internalization of rules and enactment of parentally requested behaviour than power assertion [18]. These findings are generally consistent with predictions derived from SDT, a general theory on motivation that is applied increasingly in research on parenting [20, 21]. In SDT, a distinction is made between autonomy-supportive and controlling parenting.

Autonomy-supportive parenting is characteristic of parents who promote children’s volitional functioning. They can do so by taking the child’s frame of reference, by providing a personally relevant rationale when introducing rules, and by allowing choice whenever possible [20, 22]. Of these autonomy-supportive practices, we focused on the provision of a rationale (which is similar to Hoffman’s concept of inductive discipline) because this strategy is most directly relevant in the context of parental rule-setting [23]. In contrast, controlling parents pressure the child to think, feel, or behave in particular ways. Instead of taking their child’s perspective, controlling parents pressure their children on the basis of their personal agenda. They can do so through a variety of tactics, including threats of punishment, taking away privileges, and guilt-trips [21]. In this study we focused on parents’ reliance on authority-based and externally pressuring tactics, which are similar to Hoffman’s concept of power assertion [18].

Consistent with Hoffman’s theory [18], in SDT it is assumed that an autonomy-supportive parental style fosters children’s internalization of rules [20]. When parents are perceived as autonomy-supportive, children would more easily accept parents’ rules and personally endorse those rules. In contrast, a controlling style of communication would forestall internalization of parental rules and may even elicit reactance against those rules. Research has confirmed these expectations in general [24] and within specific domains of children’s lives, including friendships and moral behaviour [25].

The current study is the first to examine the role of autonomy-supportive and controlling styles of communication in children’s screen time. Given their differential role in the process of internalization, it was expected in the current study that an autonomy-supportive style of communicating rules for screen time would relate negatively to children’s screen time while a controlling style would relate positively. We expected that parents’ style of communicating rules would have predictive value in addition to the effect of parents’ degree of regulation as such. According to SDT, autonomy-supportive and controlling parental styles should have similar effects across countries and cultures because they appeal to children’s universal psychological needs for autonomy, competence and relatedness [19]. Another way in which this study can contribute to the literature is by examining the hypotheses in a large sample of parents and children across Europe, examining the generalization of the hypothesized effects across cultures. The aims of this study were to examine associations of parental rules and parental style of communication with children’s screen time and perceived excessive screen time in five European countries.

## Methods

### Study design and participants

This study uses data from the pre-test survey (both the control and the intervention group) of the evaluation of the UP4FUN intervention, which is part of the ENERGY project (“EuropeaN Energy balance Research to prevent excessive weight Gain among Youth”-project) [26]. UP4FUN was a multi-centre, cluster randomised controlled trial with pre- and post-test measurements and an equal number of intervention and control schools in each of five countries: Belgium, Germany, Greece, Hungary and Norway. A convenience sample of schools was chosen, i.e., schools close to the research institutions. Of the 152 schools invited, 62 schools agreed to participate (participation rate 41 %). More information about the UP4FUN study has been published elsewhere [27]. A test-retest reliability study was conducted on the UP4FUN child and parent questionnaires. A convenience sample of six schools was selected in autumn 2011: one school in Belgium, four schools in Hungary and one school in Norway, including 143 pupils and 105 parents [28].

The eligible study population was the pupils from the two grade levels that included the majority of children born in the years 1999 and 2000 (i.e., children who were 10–12 years old in the autumn of 2011), and also one parent of every child. There were 5117 eligible children at the 62 schools. The pre-test data was collected in September/October 2011. Paper-and-pencil questionnaires were completed by the children within one school lesson (45 min) in the presence of project members following the UP4FUN study protocol. On the same day, the parent questionnaire was brought home for completion by one of the parents. More extensive information about the design and participants is reported elsewhere [27].

The study adhered to the Helsinki Declaration and the conventions of the Council of Europe on human rights and biomedicine. All participating countries obtained ethical clearance from the relevant ethical committees and ministries. The following ethical committees gave their approval to the study:

- Belgium: Medical Ethics Committee of the University Hospital Ghent
- Germany: State Medical Chamber of Baden-Württemberg
- Greece: Bioethics Committee of Harokopio University
- Hungary: Scientific and Ethics Committee of Health Sciences Council
- Norway: National Committees for Research Ethics in Norway.

Written parental consent was required for pupils’ participation in the study in all countries except Belgium. In Belgium, the information letters were given to the children and teachers, and the researchers highlighted that the letter had to be brought home and given to the parents. The participating parents consented by returning the parent questionnaire, as passive informed consent was allowed by the ethics committee. The study is registered in the International Standard Randomized Controlled Trial Number Register (registration number: ISRCTN34562078).

### Measurements

Screen time was assessed in terms of time spent watching TV and DVD and in terms of time spent using computer and games console. In addition to examining children’s degree of screen time as an outcome, we examined children’s excessive screen time behaviours, as perceived by both children and parents. The questionnaires was developed in English, translated into the relevant five languages, back-translated into English and checked for misinterpretation by people who were native speakers of the languages and fluent speakers of English.

#### Usual screen time

TV and DVD time was assessed by asking the children “Roughly how many hours a day do you usually spend watching TV/DVD in your leisure time?” for weekdays and weekend days separately. Computer time and time used for games console (hours/day) were assessed similarly by asking the children “Roughly how many hours a day do you usually use a computer/games console for leisure activities?” There were ten answer categories used for all four questions, ranging from “None at all” (0), “less than 30 min/day”, “30 min/day”, to “4 or more hours/day” (4) with 30 min intervals. Time spent watching “TV/DVD” was defined as watching all TV programs and films (also DVD) on a TV or on a computer. Use of “computer/games console“was defined as playing games on a computer, games console or mobile phone and using the Internet for leisure activities such as chatting, e-mailing and surfing. These questions showed good test-re-test reliability (ICCs: 0.74–0.84) [28] and moderate to good construct validity (ICCs: 0.56–0.68) in a separate study [29]. However, average computer time on a weekday had poor construct validity (ICC: 0.38) [29]. Mean scores for TV/DVD time and computer/games console time per day (hours/day) were computed.

#### Perceived excessive screen time

Perceived excessive screen time was assessed in children with the statements “I spend too much time watching TV/DVD” and “I spend too much time using a computer/games console for leisure activities”. Parents reported their perception of the child’s excessive use by the statements “My child spends too much time watching TV/DVD” and “My child spends too much time using a computer/games console for leisure activities”. The five answer categories for both children and parents ranged between “I fully agree” and “I fully disagree”, which was recoded into no excessive TV/DVD use (answer categories “I fully disagree” – “Neither agree nor disagree”) and excessive TV/DVD use (answer categories “I agree”- “I fully agree”). The test-retest reliability showed acceptable to good values for the excessive use variables reported by children and parents (0.51–0.69) [28].

#### Parental rules

Presence of parental rules was assessed in children by the statement “My parents/guardians have rules about how much I am allowed to watch TV/DVD” and “My parents/guardians have rules about how much I am allowed to use a computer/games console for leisure activities”. For parents the parallel statements were “I have rules about how much my child is allowed to watch TV/DVD” and “I have rules about how much my child is allowed to use a computer/games console for leisure activities”. The original five answer categories for both children and parents ranged from “I fully agree” to “I fully disagree”. The test-retest reliability from the study conducted on the UP4FUN child and parent questionnaires showed acceptable to good values for the rule variables reported by children and parents (0.55–0.69) [28]. As “to agree” reflects a positive response to the presence of rules and “disagree” reflects a negative answer, these categories were recoded into “Yes” (including fully/partly agree) and “No” (including neither agree nor disagree/partly disagree/fully disagree).

#### Parental style of communication

The assessment of parental style of communicating rules was based on questions and answer categories developed in previous studies [25, 30]. The psychometric properties of the original scale were found to be satisfactory [30]. In the child version of the UP4FUN questionnaire, children read the following item: “How do your parents/guardians react if you want to watch more TV/DVD than they want you to?” This item was followed by two items, one tapping into a controlling style (“They become angry and tell me to do as they say”) and one tapping into an autonomy-supportive style (“They give a clear and sensible reason as to why they do not want me to watch TV/DVD too often”). Participants provided separate ratings on each of these two items (i.e., they were not asked to make a forced choice between both items). The five answer categories for both styles ranged from “I fully agree” to “I fully disagree”. Similarly, the item for parental style related to use of computer/games console was “How do your parents/guardians react if you want to use a computer/games console more than they want you to?” This item was followed by the same two items used for parental style of communicating rules related to TV/DVD use.

In the parent questionnaire, parents received the parallel questions. For instance, for TV/DVD parents were presented with the item stem: “How do you react if your child wants to watch more TV/DVD than what you want him/her to do?” followed by one item tapping into controlling style (“I become angry and tell him/her to do as I say”) and one item tapping into autonomy-supportive style (“I give a clear and sensible reason as to why I do not want my child to watch TV/DVD too often.”). Items were rated by parents on the same scale as the one used for the children. Similarly, the item assessing parental style related to use of computer/games console was “How do you react if your child wants to use a computer/games console more than what you want him/her to do?” The test-retest reliability showed acceptable values for the parental style variables reported by children and parents (0.43–0.66) [28].

### Family educational level

Family educational level was reported by the parent filling out the parental questionnaire, and was used as an indicator of socio-economic status. One parent was asked to report on the years of education of both parents. The question was “How many years of school education did you/your partner complete?” The six answer categories ranged from “less than 7 years” to “16 years or more”. Based on preliminary analyses of the distribution of family educational level, it was decided to dichotomize the variable. The educational level was categorised as “none of the parents followed education for 14 years or more” and “one or both parents followed education for 14 years or more”.

Due to missing values in the parental education variable, the number of parents included in some of the analyses is lower than the total number of parents participating in the study, which will be indicated in the relevant tables.

### Statistical analysis

Predictive Analytics SoftWare (IBM SPSS Statistics for Windows, Version 22.0. Armonk, NY: IBM Corp.) was used to perform the descriptive analyses and to calculate the Intra Class Correlation (ICC) for potential clustering effects due to the nested design. These clustering effects of children nested in schools were checked by the Linear Mixed Model procedure. No clustering effect was found for the adolescents’ time used for TV/DVD and computer/games console and only 3 % (TV/DVD) and 7 % (computer/games console) of the unexplained variation was at the group level [31]. Because there was no substantial clustering effect, it was not taken into account in the analyses. In initial analyses (data not shown), the correlation between child reported TV/DVD time and perceived excessive TV/DVD time was 0.33, and between child reported computer/games console time and perceived excessive computer/games console time the correlation was 0.44. The correlation between child reported perceived excessive TV/DVD time and parent reported perceived excessive TV/DVD time was 0.26 in initial analyses. The exact agreement was 29.2 % (data not shown). About the same correlation was found for child reported perceived excessive computer/games console time and parent reported perceived excessive computer/games console time (0.29). The exact agreement was 32.9 % (data not shown). The parents were not asked questions about how much time the children spend on TV/DVD and computer/games console, so no correlations could be calculated between children’s screen time and perceived excessive screen time for TV/DVD and computer/game consoles reported by parent. Due to the weak to moderate correlations, separate models were run for actual and perceived screen time, and for parent and child reports.

The data are presented as means (standard deviation; SD) or percentages, unless stated otherwise.

Associations of rules and parenting style with usual screen time and perceived excessive screen time were estimated in Mplus (version 5, Muthen & Muthen). Three structural equation models were tested when exploring the associations between child and parent reported rules and parenting style with child reported screen time; Model 1 included a path from rules to screen time adjusted for country, age and sex; Model 2 included rules and style as simultaneous predictors of screen time (adjusted for country, age and sex); and Model 3 was the same as Model 2, but additionally included parental education as a control variable. To assess whether associations between independent and dependent variables were country-specific, we estimated multi group models (for models 1, 2 and 3) and compared these with the total group models using the difference in Chi square to test significant improvement in model fit. Other fit indexes such as TLI, CFI, RMSE, BIC, and AIC were also used to judge model fit. Comparison of models with dichotomous outcomes for the total sample with country-specific models (TYPE = MIXTURE) was done using the Sattora-Bentler Scaled Chi-Square test (Χ_SB_^2^) [32]. Country-specific results are presented in those cases where the multi group models fitted better than the total group models. Statistical significance was set at *P* < 0.05.

## Results

Parents of 3394 children (66 %) provided parental consent for study participation, and a total of 3325 children and 3038 parents (81 % mothers) completed the pre-test questionnaires. The sample consisted of 11-year-old children (average age 11.2 years and 51 % girls) and one of their parents (81 % mothers). The parental educational level was high; in 64 % of the families at least one of the parents had 14 years or more of education.

### Screen time

The average TV/DVD times in the five countries were between 1.5 and 1.8 h (Table 1). The children in Greece spent most time in front of TV/DVD (1.8 (SD 1.0) hours/day) while the Norwegian and German children spent least time watching TV/DVD (1.5 (SD 0.98/0.94) hours/day). Less time was used for computer/games console in the five countries (about 0.9–1.4 h). Children in Hungary spent most time on computer/games console (1.4 (SD 1.2) hours/day) while Belgian children spent least time (0.9 (SD 0.9) hours/day) (Table 1). For the total sample, 37.5 % of the children reported that they spend too much time on TV/DVD and 29.5 % reported that they spend too much time on computer/games console. The parallel proportions of the parents who thought their child spend too much time in front of screens were 41.6 and 36.8 %, respectively. There were more pronounced between-country differences in perceived excessive screen time than in screen time per se. More than 50 % of both children and parents reported that parents have rules about how much time the child was allowed to use for watching TV/DVD and using the computer/games consoles.

**Table 1:** Characteristics (demographic and behaviours) for total sample and by country

	Belgium		Germany		Greece		Hungary		Norway		Total sample	
	*N* = 728		*N* = 609		*N* = 722		*N* = 658		*N* = 583		*N* = 3300	
	Mean	(SD)	Mean	(SD)	Mean	(SD)	Mean	(SD)	Mean	(SD)	Mean	(SD)
Age	10.9	(0.71)	11.2	(0.77)	10.8	(0.63)	11.6	(0.69)	11.9	(0.57)	11.2	(0.78)
Gender, children (%)												
Girl	52.1		47.9		54.7		50.3		50.3		51.2	
Gender, parents (%)												
Mother	81.9		80.9		80.7		87.2		74.9		81.3	
Family educational level;												
At least one parent ≥ 14 years of education (%)	88.1		47.9		61.7		48.6		71.4		63.7	
	Mean	(SD)	Mean	(SD)	Mean	(SD)	Mean	(SD)	Mean	(SD)	Mean	(SD)
Time spent watching TV/DVD in leisure time, hours/day	1.55	(0.93)	1.53	(0.98)	1.84	(1.01)	1.69	(1.09)	1.53	(0.94)	1.63	(1.00)
Time spent using a computer/games console for leisure activities, hours/day	0.93	(0.90)	1.04	(0.99)	1.24	(1.01)	1.36	(1.16)	1.30	(1.02)	1.17	(1.03)
Child reported perceived excessive screen time (%)^1^												
- TV/DVD	57.4		25.5		26.0		42.3		33.8		37.5	
- Computer/games console	36.1		15.4		28.3		35.0		31.3		29.5	
Parent reported perceived excessive screen time (%)^1^												
- TV/DVD	50.3		34.1		35.4		44.9		42.2		41.6	
- Computer/games console	38.8		24.7		33.3		42.3		45.6		36.8	
Rules present												
- TV/DVD: Yes	55.6		48.4		64.1		58.7		39.8		54.0	
- Computer/games console: Yes	53.4		43.8		66.1		66.2		43.9		55.3	

*SD* standard deviation

N vary slightly, n for gender is presented

^1^Fully and partly agree that child spends too much time in front of screen, combined

### Parental style

In the total sample, the children’s perceived parental style of communication was quite consistent for TV/DVD and computer/games console. Approximately 40 % of the children fully or partly agreed with statements reflecting a controlling style for communicating rules related to screen time, while about 70 % agreed fully or partly with statements reflecting an autonomy-supportive style. There was some variance between countries, with the lowest proportions of full agreement with a controlling style (8–9 %) and with an autonomy-supportive style (26 %) observed in the Norwegian children. The tendencies were the same for the parent reported style of communication; close to 50 % of the parents fully or partly agreed with statements reflecting a controlling style, while about 90 % agreed fully or partly with statements reflecting an autonomy-supportive style. Children and parents disagreed most with regard to the endorsement of an autonomy-supportive style, with parents reporting higher levels of this style than children. Again, the lowest proportions of full agreement with a controlling (11–12 %) and with an autonomy-supportive style (48–50 %) were observed in Norwegian parents (Table 2).

**Table 2:** Perceived parental style, reported by children and parents, total sample and by country

Parental style	Belgium		Germany		Greece		Hungary		Norway		Total	
Reported by:	Child	Parent	Child	Parent	Child	Parent	Child	Parent	Child	Parent	Child	Parent
TV/DVD												
Controlling; (N)	725	593	580	474	722	614	656	592	578	464	3261	2737
I fully agree (%)	22.3	19.2	20.9	19.4	18.6	22.5	23.9	25.8	8.0	11.1	19.0	20.0
I partly agree (%)	33.5	27.5	16.2	25.9	23.0	23.9	23.3	31.4	21.3	34.9	23.9	28.5
Neither nor (%)	17.4	17.4	26.0	18.1	24.2	13.4	16.5	5.7	31.8	19.6	22.8	14.5
I partly disagree (%)	9.7	11.8	12.4	14.1	13.9	18.7	11.7	13.3	17.1	14.9	12.8	14.6
I fully disagree (%)	17.1	24.1	24.5	22.4	20.4	21.5	24.5	23.6	21.8	19.6	21.5	22.4
Autonomy-supportive (N)	722	616	595	512	721	653	654	617	576	476	3268	2874
I fully agree (%)	55.4	66.2	47.4	70.3	61.6	82.8	52.4	77.8	26.4	49.8	49.6	70.5
I partly agree (%)	23.8	23.7	21.3	20.3	15.1	12.7	16.5	16.2	27.3	37.0	20.6	21.2
Neither nor (%)	8.2	5.2	15.5	5.5	12.6	2.3	11.9	2.4	24.1	10.1	14.0	4.8
I partly disagree (%)	4.7	1.5	5.4	2.1	3.2	1.5	4.7	1.6	10.2	1.9	5.5	1.7
I fully disagree (%)	7.9	3.4	10.4	1.8	7.5	0.6	14.4	1.9	12.0	1.3	10.3	1.8
Computer/games console												
Controlling (N)	716	591	573	473	721	608	652	581	571	457	3233	2710
I fully agree (%)	22.8	18.3	19.7	18.6	18.9	22.9	21.2	25.0	8.8	12.9	18.6	19.9
I partly agree (%)	26.3	25.4	14.0	24.7	17.5	25.8	23.5	28.2	21.4	34.1	20.7	27.5
Neither nor (%)	20.1	21.0	24.6	16.5	26.8	10.5	15.5	6.0	29.8	20.1	23.2	14.5
I partly disagree (%)	10.8	9.3	11.5	14.6	12.2	21.1	13.2	13.3	16.5	12.0	12.7	14.2
I fully disagree (%)	20.1	26.1	30.2	25.6	24.7	19.7	26.7	27.5	23.6	20.8	24.9	24.0
Autonomy-supportive (N)	715	615	590	514	719	645	655	608	578	478	3257	2860
I fully agree (%)	52.9	66.7	43.6	68.1	61.9	83.3	49.8	77.8	26.5	47.7	47.9	69.9
I partly agree (%)	23.4	22.4	15.6	21.0	15.4	12.7	22.3	14.1	28.4	37.4	20.9	20.7
Neither nor (%)	10.2	7.0	18.8	6.8	13.9	2.9	11.9	3.0	23.9	10.7	15.4	5.8
I partly disagree (%)	2.5	1.6	6.4	2.5	2.6	0.5	4.6	3.3	9.7	2.7	4.9	2.1
I fully disagree (%)	11.0	2.3	15.6	1.6	6.1	0.6	11.5	1.8	11.6	1.5	11.0	1.5

### Associations between rules, parenting style and screen time

When estimating Model 3 (Table 3), the presence of rules was significantly associated with less time watching TV/DVD, both according to data from children and parents (child reported estimate = −0.112 (−0.149; −0.075) and parent reported estimate = −0.091 (CI −0.128; −0.054) hours/day, which equals about 12 min per day). For computer/games console the presence of rules was significantly associated with less time, according to the child data (child reported estimate = −0.067 (CI −0.104;-0.030) hours/day, which equals about 8 min per day). In addition to the setting of rules per se, the use of an autonomy-supportive style was related negatively to both TV/DVD viewing and use of computer/games console, according to the child data (about 3 min, estimate TV/DVD = −0.07 (CI −0.109; −0.031), estimate computer/games console = −0.06 (CI −0.099; −0.021)). Based on the parental data, the use of a controlling style was related positively to time used for computer/games console (about 5 min, estimate = 0.12 (CI 0.085; 0.155)).

**Table 3:** Associations between screen time and parental rules/style reported by children and parents, in total sample

Outcome	Model 1		Model 2		Model 3	
	Standardized B	95 % CI	Standardized B	95 % CI	Standardized B	95 % CI
TV/DVD (hrs/day), child reported						
Rules (yes vs no)	**−0.126**	**(−0.159; −0.093)**	**−0.101**	**(−0.136; −0.066)**	**−0.112**	**(−0.149; −0.075)**
Controlling style	-	-	0.004	(−0.031; 0.039)	−0.007	(−0.044; 0.030)
Autonomy-supportive style	-	-	**−0.084**	**(−0.121; −0.047)**	**−0.07**	**(−0.109; −0.031)**
Computer/game consoles (hrs/day), child reported						
Rules (yes vs no)	**−0.09**	**(−0.123; −0.057)**	**−0.071**	**(−0.106; −0.036)**	**−0.067**	**(−0.104;-0.030)**
Controlling style	-	-	**0.038**	**(0.003; 0.073)**	0.031	(−0.006;0.068)
Autonomy-supportive style	-	-	**−0.077**	**(−0.112; −0.042)**	**−0.06**	**(−0.099; −0.021)**
TV/DVD (hrs/day), parent reported						
Rules (yes vs no)	**−0.10**	**(−0.137; −0.063)**	**−0.093**	**(−0.130; −0.056)**	**−0.091**	**(−0.128; −0.054)**
Controlling style			−0.033	(−0.070; 0.004)	−0.032	(−0.069; 0.005)
Autonomy-supportive style			0.012	(−0.025; 0.049)	0.01	(−0.027; 0.047)
Computer/games console (hrs/day), parent reported						
Rules (yes vs no)	−0.03	(−0.067; 0.007)	−0.028	(−0.067; 0.011)	−0.025	(−0.064; 0.014)
Controlling style			**0.13**	**(0.095; 0.165)**	**0.12**	**(0.085; 0.155)**
Autonomy-supportive style			**−0.041**	**(−0.078; −0.004)**	−0.038	(−0.075; −0.001)
	OR	95 % CI	OR	95 % CI	OR	95%CI
*Perceived excessive* TV/DVD time, child reported (dichotomous)						
Rules (yes vs no)	0.922	(0.786; 1.081)	0.973	(0.820; 1.153)	0.999	(0.841; 1.186)
Controlling style			**1.146**	**(1.079; 1.216)**	**1.142**	**(1.076; 1.213)**
Autonomy-supportive style			0.996	(0.933; 1.064)	1.004	(0.940; 1.073)
*Perceived excessive* computer/games console time, child reported (dichotomous)						
Rules (yes vs no)	**1.218**	**(1.025; 1.446)**	1.102	(0.918; 1.324)	1.102	(0.918; 1.324)
Controlling style			**1.201**	**(1.130; 1.276)**	**1.200**	**(1.129; 1.275)**
Autonomy-supportive style			1.017	(0.949; 1.091)	1.022	(0.952; 1.096)
*Perceived excessive* TV/DVD time, parent reported (dichotomous)						
Rules (yes vs no)	**0.192**	**(1.016; 1.445)**	1.183	(0.974; 1.437)	1.196	(0.983; 1.455)
Controlling style			**1.354**	**(1.280; 1.433)**	**1.349**	**(1.274; 1.427)**
Autonomy-supportive style			0.970	(0.878; 1.071)	0.978	(0.885; 1.081)
*Perceived excessive* computer/games console time, parent reported (dichotomous)						
Rules (yes vs no)	1.430	(1.171; 1.747)	**1.406**	**(1.123; 1.760)**	**1.395**	**(1.112; 1.748)**
Controlling style			**1.341**	**(1.266; 1.420)**	**1.339**	**(1.264; 1.419)**
Autonomy-supportive style			0.977	(0.874; 1.091)	0.975	(0.872; 1.091)

*B* regression coefficient, *OR* odds ratio, *95 % CI* 95 % confidence interval

Model 1 – adjusted for country, age and sex

Model 2 – Model 1 + styles included

Model 3 – Model 2 + further adjusted for education

Bold = significant

### Associations between rules, parenting style and perceived excessive screen time

The presence of rules was positively related to perceived excessive computer/games console time according to data from parents (OR = 1.4 (CI 1.112; 1.748)) (Table 3). Associations between a controlling style and perceived excessive screen time were quite consistent, with the use of a controlling style being related positively to time used on TV/DVD (Odds ratio OR = 1.3 (CI 1.274; 1.427), parental data) and computer/games console (OR = 1.2 (CI 1.129; 1.275), child data and OR = 1.3 (CI 1.264; 1.419), parental data).

### Country specific associations between rules, parenting style and (perceived excessive) screen time

For the child reported computer time and for the child reported perceived excessive computer time, the models specifying country specific associations fitted better than the models assuming the same associations across countries (*Χ*^2^ = 296.0, df = 30, *p* = 0.0276 and Χ_SB_^2^ = 56.57, df = 24, *p* < 0.001 respectively). For these two outcome variables, the country specific results are presented in Table 4. In two countries the presence of rules was significantly associated with less time used for computer/games console, meaning about 16 min (child reported estimate = −0.131 (CI −0.207; −0.055)) in Greece and about 12 min (child reported estimate = −0.085 (CI −0.167; −0.003)) in Hungary (Table 4). The use of a controlling style was related positively to time used for computer/games console in Greece and Norway (about 4–5 min more, child reported estimate Greece = 0.08 (CI 0.007; 0.153) and child reported estimate Norway = 0.1 (CI 0.010; 0.190)). The use of an autonomy-supportive style was related negatively to use of computer/games console in Greece and Germany (about 4 min less, child reported estimate Greece = − 0.092 (CI −0.168; −0.016) and child reported estimate Germany = −0.107 (CI −0.201; −0.013)). The presence of rules was positively related to perceived excessive computer/games console time according to data from children in Greece, Norway and Germany (OR = 1.3–1.7) (Table 4). A controlling style was related positively to perceived excessive time used on computer/games console, based on child data from Belgium, Hungary, Norway and Germany (OR = 1.3–1.4), while the ratio for Greece was OR = 0.74 (CI 0.55; 1.00).

**Table 4:** Country-specific results from multi group analyses; associations between parental style of rules and screen time

	Belgium		Greece		Hungary		Norway		Germany	
	B ^1^	95 % CI	B ^1^	95 % CI	B ^1^	95 % CI	B ^1^	95 % CI	B ^1^	95 % CI
Computer/games console, child reported (hrs/day)										
Rules (yes vs no)	−0.008	(−0.092; 0.076)	**−0.131**	**(−0.207; −0.055)**	**−0.085**	**(−0.167; −0.003)**	−0.018	(−0.110; 0.074)	−0.068	(−0.158; 0.022)
Controlling style	0.002	(−0.082; 0.086)	**0.08**	**(0.007; 0.153)**	−0.03	(−0.108; 0.048)	**0.1**	**(0.010; 0.190)**	0.015	(−0.075; 0.105)
Autonomy-supportive style	−0.054	(−0.138;0.030)	**−0.092**	**(−0.168; −0.016)**	−0.006	(−0.088; 0.076)	−0.063	(−0.157; 0.031)	**−0.107**	**(−0.201; −0.013)**
	OR	95 % CI	OR	95 % CI	OR	95 % CI	OR	95 % CI	OR	95 % CI
*Perceived excessive* computer/games console time, child reported (dichotomous)										
Rules (yes vs no)	1.38	(0.90; 2.12)	**1.33**	**(1.17; 1.51)**	1.07	(0.59; 1.96)	**1.65**	**(1.06; 2.57)**	**1.69**	**(0.94;3.03)**
Controlling style	**1.37**	**(1.21; 1.55)**	**0.74**	**(0.55; 1.00)**	**1.35**	**(1.20;1.51)**	**1.27**	**(1.10; 1.46)**	**1.39**	**(1.19;1.62)**
Autonomy-supportive style	1.17	(0.93; 1.46)	1.20	(0.83;1.73)	0.97	(0.77;1.22)	1.03	(0.80;1.33)	0.90	(0.70;1.14)

*B* Standardized regression coefficient, *OR* odds ratio, *95 % CI* 95 % confidence interval

^1^ analyses are adjusted for sex, age and educational level

Bold = significant

## Discussion

This study showed that the average time used for TV/DVD and computer/games control is rather similar across European 10–12-year-olds, while the proportion of children reporting that they spend too much time on TV/DVD and computer/games console varies between the countries (TV/DVD: 25.5–57.4 %, computer/games console: 15.4–36.1 %). The children’s perceived parental style of communication was quite consistent for TV/DVD and computer/games console. The presence of rules was significantly associated with less time watching TV/DVD and use of computer/games console time. Moreover, the use of an autonomy-supportive style was related negatively to both time watching TV/DVD and use of computer/games console time. The use of a controlling style was related positively to perceived excessive time used on TV/DVD and excessive time used on computer/games console. With a few exceptions, results were similar across the five countries. These results suggest that parental style is important in addition to the presence of rules.

The time used for TV/DVD and computer/games console in the children is in accordance with the levels reported in the ENERGY cross sectional study; on average 1.63 h/day in all countries for TV/DVD and 1.17 for computer/games console activities [4]. In the total sample, 30–40 % of children and parents agreed in perceiving that the child spends too much time in watching TV/DVD and using a computer/games console. Barr-Anderson et al. [33] reported that 23 % of parents (children’s mean age 5.8 years) strongly agreed or agreed that their child spent too much time watching TV, and 15 % (strongly) agreed that their child spent too much time playing video games. Our results are in accordance with these results, finding that a higher proportion of parents reported perceived excessive TV time compared to time used on computer/games console. The more pronounced between-country differences in perceived excessive screen time than in screen time per se may be due to differences between countries in cultural norms regarding appropriate amounts of screen time. Te Velde et al. [34] indeed reported a wide variation in parental norms for TV viewing across Europe, meaning that in some countries children report positive parental norms regarding TV viewing, which may indicate that parents in these countries are not too concerned about TV-time.

Consistent with several previous studies and reviews [9–14], presence of rules was significantly associated with less screen time in this study. At first sight, this finding seems to indicate that the communication of rules regarding screen time is adaptive and helps to reduce children’s actual screen time. However, presence of rules was also associated positively (rather than negatively) with parent reported perceived excessive computer/games console time. In three countries in particular (Greece, Norway and Germany) presence of rules was associated positively with child reported perceived excessive computer/games console time. It seems more likely, therefore, that parental rules represent reactive behaviour of parents. That is, parents would respond to children’s behaviour rather than pro-actively influencing the child’s behaviour. If the child does not watch TV a lot, rules may not be needed (resulting in a negative association between rules and screen time as such). However, when a child displays perceived excessive screen time, parents may feel a strong need to reduce screen time. As a result, they may introduce more rules and regulations (resulting in a positive association between rules and perceived excessive screen time). Future longitudinal research is needed to determine the direction of effects in associations between parental rules and screen time. The complex association between rule-setting as such and screen time also suggests the need to take into account parents’ style of introducing rules and regulating children’s screen time.

As expected on the basis of Hoffman [18] and SDT [20], an autonomy-supportive style was related negatively to screen time, at least when using child reported measures of parental style. According to the child data, these findings are consistent for both watching TV/DVD and using computer/games console. When children perceive their parents as using a more autonomy-supportive style, they spend less time in front of TV/DVD and engage less often in activities with computer/games. One interpretation of this finding is that children who perceive their parents as autonomy-supportive internalize their parents’ rules regarding screen time more easily, such that they stick to those rules more often [20, 25]. An alternative interpretation is that it is easier for parents to discuss rules regarding screen time in an autonomy-supportive fashion when children are less inclined by themselves to spend a lot of time watching TV or playing games.

Consistent with important theories of socialization such as Hoffman and SDT, a controlling style was related to more maladaptive outcomes. Both in the total sample and within each country, a controlling style was related positively to perceived excessive screen time. This association was obtained irrespective of who reported on parental style or perceived excessive screen time, and was the most robust and consistent association obtained in this study. On the basis of SDT it can be argued that a controlling parental style forestalls internalization and may even elicit psychological reactance in children, such that they watch TV and play computer games to a disproportionate and excessive degree [24, 25]. As with the other associations obtained in this study, the alternative direction of effects may also apply: when children spend excessive amounts of time in front of the TV or computer, parents may become worried [13, 35]. Negative emotions such as worry and anxiety have been shown to elicit a more controlling parental approach [36]. Longitudinal research is needed to document the undoubtedly complex and transactional associations between parental behaviour and children’s screen time. Ideally, such research would also include measures of mediating processes that may account for both parent on child effects (e.g., internalization and reactance) and child on parent effects (e.g., negative parental emotions). Finally, from whom and how to measure excessive screen time – subjectively (perceived by children and/or parents) versus objectively – need to be explored further. In this study the correlation between child and parent perceived excessive screen time was low (0.3), which might be explained by the different beliefs that parents and children have regarding what is excessive. Pearson et al. [13] report findings suggesting that parents of children aged 5–6 years and 10–12 years recognise excessive TV viewing in their children.

With a few exceptions, associations between parental style of communication and the outcomes were similar across the European countries included in this study. As such, the findings suggest that culture does not have a substantial impact on the dynamics involved in autonomy-supportive and controlling parenting as argued by Soenens and Beyers [37] and Pomerantz and Wang [38]. One notable and unexpected finding in the country-specific analyses was that a controlling style had a negative association with perceived excessive computer time in Greece. At first sight, this finding suggests that in Greece a controlling style might be an effective parental style. It could also be the case, however, that Greek participants were less inclined to interpret the items for a controlling style as representing a really pressuring style (i.e., a style thwarting children’s need for autonomy). Instead, Greek participants may have been relatively more likely to perceive the controlling approach as the provision of structure and clarity (i.e., a style that would support children’s need for competence). Another interpretation of this unexpected finding is that Greek participants had a different understanding of what excessive computer time means. Possibly, excessive computer time was less problematic in their view. This issue needs to be addressed in future research.

Furthermore, it may be questioned whether the associations found are large enough to have any public health impact. One review suggests that in children an imbalance over time of about 2 % (125 KJ or 15 min of play replaced by TV-viewing) may lead to obesity [39]. This indicates that 12 min decrease in sedentary behaviour, as found for the presence of rules related to watching TV/DVD in the total sample, may have a possible public health impact. However, this scenario requires that a potential decrease in screen time is replaced by physical activity, and not by another sedentary behaviour. Moreover, behavioural research in children shows clustering of dietary and activity behaviours in healthy (e.g., sporty-healthy eating) and unhealthy (e.g., sedentary-snacking) patterns [40]. If interventions promoting rule setting and supportive parenting styles are broadly implemented, they can have a significant impact also on other health behaviours [41]. This means that interventions like this can have a public health impact.

Although both children and parents reported that an autonomy-supportive style is used more often than a controlling style, parents (compared to children) reported higher levels of an autonomy-supportive style in particular. This result is consistent with research conducted by Gaylord et al. [42], finding that parents perceived themselves as more supportive than their 9-year-old children perceived them to be. This may indicate a higher degree of social desirability in the answers from the parents (mainly mothers), or it may indicate that there is actually a difference in perceived parenting style by children compared to the experienced parenting style in parents/mothers. Rebholz et al. [43] found that parents and children in the ENERGY study perceived parental practices regarding sedentary behaviours differently in all parts of Europe. Moreover, De Bourdeaudhuij and van Oost [44] found that parents scored higher on social desirability than children, which may lead parents to report better than what is the reality.

To our knowledge, this is the first study examining associations between parental styles of communication and screen time across Europe. Strengths of the study are the sample including both children and parents from five different European countries, and the investigation of parenting styles both for TV/DVD and computer/games console separately – reported by children as well as parents. However, there are several limitations of this study. First, when interpreting the findings we need to take into account that the study had a cross-sectional design. Therefore, no causal inferences can be drawn. Moreover, the generalizability of our findings may be limited because a convenience sample of schools was chosen, and the recruitment of schools and participants may have caused a non-response bias, restricting the range of values on our parental style measures. Furthermore, fewer parents responded to the questionnaires than children, resulting in missing values for the parental educational level variable. In addition, screen time and parental style were self-reported and might be responded to in a socially desirable way. Another limitation is that most concepts (including parental style of communication) were measured using single items. In addition to an increased likelihood that measurement error affected some of the results; the use of single items hinders a comprehensive assessment of underlying constructs. The concepts of autonomy-supportive and controlling parenting are relatively broad. In this study we measured only specific features of both styles that seemed most relevant in the context of parental rule-setting (i.e., the provision of a rationale and the use of externally pressuring language). Future research would do well to measure also other features of both styles (e.g., perspective taking and the use of internally pressuring tactics) so as to arrive at a more comprehensive assessment. Additionally, the validity of the measures used assessing parental style of communication has not been assessed, which is a limitation in this study. Finally, because most of the parents participating were mothers our data provide limited insight into the role of fathers.

## Conclusions

As hypothesized by SDT, this study suggests that an autonomy-supportive style of communicating rules regarding TV/DVD or computer use is related negatively to children’s screen time. In contrast, a controlling style is associated with more screen time and with more perceived excessive screen time in particular. Rules as such were related to both positive and negative outcomes, and the autonomy-supportive and controlling styles of communication displayed a more differentiated pattern of associations with positive and negative outcomes, respectively. With a few exceptions, these associations were consistent across 5 different European countries with one important exception to the pattern (i.e., the negative association between a controlling style and excessive computer time in Greece). The latter result needs to be examined further. Longitudinal research and research using more elaborate, multi-item measures of the communication style is needed, as well as future work exploring how to best deliver restriction messages.

## Abbreviations

SDT: Self-Determination Theory
